# Particulate matter may have a limited influence on maternal vitamin D levels

**DOI:** 10.1038/s41598-022-21383-1

**Published:** 2022-10-07

**Authors:** Chong Li, Ya-qin Gong, Yun-yu Xia, Xiao-chun Wang, Lin Chen, Shan-jun Yan, Rong-zhu Lu, Ke Lu

**Affiliations:** 1 Department of Orthopedics, Affiliated Kunshan Hospital of Jiangsu University, No. 91 West of Qianjin Road, Suzhou, 215300 Jiangsu China; 2 Information Department, Affiliated Kunshan Hospital of Jiangsu University, Suzhou, 215300 Jiangsu China; 3 Meteorological Bureau of Kunshan City, Suzhou, 215337 Jiangsu China; 4 Ecology and Environment Bureau of Kunshan City, Suzhou, 215300 Jiangsu China; 5 Department of Preventive Medicine and Public Health Laboratory Science, School of Medicine, Jiangsu University, 301 Xuefu Road, Zhenjiang, 212013 Jiangsu China

**Keywords:** Endocrine system and metabolic diseases, Environmental impact, Nutrition, Epidemiology

## Abstract

Evidence for an association between the amount of particulate matter (PM) in the atmosphere and vitamin D status of pregnant women is limited. We aimed to examine the independent association between PM and maternal levels of serum 25-hydroxyvitamin D (25OHD) during the second trimester and to explore possible modifications to the association by meteorological factors. 27,768 pregnant women presenting for prenatal examination who were tested for serum 25OHD concentration during the second trimester between January 1, 2016, and December 31, 2020, were included in this retrospective analysis. Exposure to PM was evaluated based on daily average PM with an aerodynamic diameter of ≤ 2.5 μm (PM_2.5_) and PM with an aerodynamic diameter of ≤ 10 μm (PM_10_). Corresponding meteorological data for daily average atmospheric temperature, atmospheric pressure, relative humidity, sunshine duration, and wind speed were collected. The maximum cumulative effects of PM_2.5_ occurred at lag 45 days, and the maximum cumulative effects of PM_10_ occurred at lag 60 days. In crude models, 45-day moving daily average PM_2.5_ concentrations were negatively associated with 25OHD levels (β, − 0.20; 95% CI − 0.21 to − 0.19), as were 60-day moving daily average PM_10_ concentrations (β, − 0.14; 95% CI − 0.15 to − 0.14). After adjusting for temporal and meteorological factors, the effect values were drastically reduced (adjusted β of PM_2.5_, − 0.032; 95% CI − 0.046 to − 0.018; adjusted β of PM_10_, − 0.039; 95% CI − 0.049 to − 0.028). Our study showed there was a small, independent, negative association between PM in the atmosphere and maternal serum 25OHD levels during the second trimester of pregnancy after adjusting for temporal and/or meteorological factors, which indicates that PM may have a limited influence on maternal serum 25OHD levels. Besides taking vitamin D supplements, pregnant women should keep participating in outdoor activities while taking PM protection measures to improve their vitamin D levels when PM levels are high in winter and spring.

## Introduction

The key fat-soluble nutrient vitamin D has multiple functions, and its deficiency is thought to be a risk factor in skeletal health and various non-skeletal conditions, such as schizophrenia, skin disorders, certain types of cancer, type 2 diabetes, and infections^1^. For pregnant women, sufficient vitamin D stores need to be maintained to provide for both themselves and their fetus^2^. Research continues to provide evidence of an association between maternal vitamin D deficiency (VDD) and a higher risk of numerous undesirable pregnancy outcomes, such as a low body weight, respiratory tract infections, and neurocognitive developmental problems in newborns and preeclampsia and high blood pressure in mothers^3–8^. Therefore, VDD is a key clinical issue for pregnant women and deserves research attention. Throughout the world at present, VDD during pregnancy is still a frequent occurrence. In one study of pregnant women in Finland, 77.4% were found to suffer from VDD (25-hydroxyvitamin D [25OHD] < 50 nmol/L) during their first trimester^9^. While in, the USA, Ginde et al.^10^ reported 46%, 32%, and 18% of women with VDD (25OHD < 50 nmol/L) in the first, second, and third trimesters, respectively, and there were comparable observations among British^11^, Indian^12^, and Chinese^13^ women.

For the most part, factors determining the vitamin D status during pregnancy do not differ from those important for the general population, such as the number of melanocytes in the skin, the amount of exposure of the skin to the sun, the amount of adipose tissue in the body, geographical latitude, dietary factors, and intake of supplementary vitamins^14–18^. An indoor lifestyle can also increase the risk of developing a VDD^19^. Moreover, the amount of PM in the atmosphere has been associated with circulating levels of 25OHD in humans^20–23^, including pregnant women^24^.

It is not yet clear what mechanisms coordinate the links between maternal vitamin D and air pollution levels. Because vitamin D is a relatively scarce resource in food, humans generally rely on the vitamin D produced by cells in the skin on exposure to natural solar ultraviolet (UV) B radiation^25,26^. Some scholars have hypothesized that the reduction in the amount of solar UVB radiation reaching ground level by particulate matter (PM) is the main mechanism linking PM to circulating 25OHD levels in humans^23,24^. In an investigation into the relationship between certain pollutants and ground-level UVB intensity, PM with an aerodynamic diameter of ≤ 10 μm (PM_10_) was shown to be a significant negative predictor of solar UVB radiation, but the impact of PM_10_ was very small^27^. In addition, vitamin D status correlated with temporal and meteorological factors. During their second trimester, the 25OHD levels of women in eastern China demonstrated variation with season and air temperature^28^. Another study reported 25OHD levels were positively correlated with the prior month’s temperature^29^. Thus, when exploring the independent association between PM and vitamin D status, meteorological factors may need to be taken into account.

Thus, our study objective was to examine the independent association between PM and maternal serum 25OHD levels during the second trimester in pregnant women and to explore if meteorological factors have an impact on this association.

## Methods

### Study population

We designed a single-hospital-based cross-sectional retrospective observational study to test our hypotheses. All prenatal examination data were collected from the hospital medical database in Affiliated Kunshan Hospital of Jiangsu University located in Kunshan, eastern China, at 31.2° N latitude and approximately 30 km from Shanghai. This is the largest hospital that provides prenatal care in all the districts of Kunshan. Consecutively, 35,476 pregnant women at the 15–20th week gestation (during the second trimester) who visited our institution for prenatal examination and were tested for serum 25OHD concentrations between January 1, 2016, and December 31, 2020, were included in this study. We excluded 7708 women for the following reasons: those (1) with a chronic metabolic disease with consequences for the metabolism of vitamin D (n = 5389); (2) with a high-risk pregnancy (n = 1087), or (3) who had lived in Kunshan for less than 1 year (n = 1232). Finally, 27,768 pregnant women were included in this analysis (Fig. S1).

The study protocol, submitted for review by the ethical committee at the Affiliated Kunshan Hospital of Jiangsu University (approval No. 2020-03-046-K01), was approved, and it complied with the Declaration of Helsinki. Patient information was initially documented for hospital’s quality improvement purposes. The blood samples used for measuring 25OHD were taken as part of prenatal examinations. The requirement for informed consent was waived because of the anonymous and observational design of this investigation. Data analyzers were blinded to the identity of the patients.

### Measurement of vitamin D

In humans, the most abundant form of vitamin D in the blood is 25OHD, and serum levels are used to reliably estimate a patient’s vitamin D status. All pregnant women in this study were in their 15–20th week of gestation (during the second trimester of pregnancy) and were asked to fast before blood samples were taken. Serum 25OHD concentrations were measured immediately using an automated electrochemiluminescence immunoassay on a Roche Cobas 8000/e602 analyzer (Roche Diagnostics, Mannheim, Germany). There is no medical consensus regarding the status cut-off values of 25OHD concentrations. However, the Institute of Medicine (National Academy of Sciences, Washington, DC, USA) and the National Osteoporosis Society (Bath, England) concur that, for bone health, a serum 25OHD level (2.5 nmol/L 25OHD = 1 ng/mL 25OHD) of < 30 nmol/L (12 ng/mL) is deficient, 30–50 nmol/L (12–20 ng/mL) is insufficient for certain individuals, while > 50 nmol/L (20 ng/mL) is sufficient for most people^30,31^. The time of blood collection was included in the analysis. Seasons were defined as: Spring, March–May; Summer, June–August; Autumn, September–November; Winter, December–February.

### PM exposure and meteorological data assessment

We received data for the daily average concentration of PM with an aerodynamic diameter of ≤ 2.5 μm (PM_2.5_; μg/m^3^) and PM_10_ (μg/m^3^) from the Meteorological Bureau of Kunshan City. Meteorological data (daily average atmospheric temperature [°C], atmospheric pressure [hPa], relative humidity [%], sunshine duration [hour], and wind speed [m/s]) were obtained from the Ecology and Environment Bureau of Kunshan City. The distances between the three environmental (No. 2 Middle School monitoring site, Zhenchuan Middle School monitoring site, and Kunshan Deng-yun College monitoring site) and one meteorological (National Meteorological Observing Station) monitoring site and the hospital are within 11 km. Kunshan City covers an area of 927.7 square kilometers; this distance is shorter than that used for sensitivity analysis in the study by Di et al. in 2017^32^.

Because the serum 25OHD half-life is about 15 days^33^, the cumulative effects of PM_2.5_ and PM_10_ on maternal serum 25OHD levels during the second trimester of pregnancy were approximated using the moving-average lag structures (blood collection day [day 0] was not included) as follows: 0–3 days (3-day moving daily average PM concentration), 0–7 days (7-day moving daily average PM concentration), 0–15 days (15-day moving daily average PM concentration), 0–30 days (30-day moving daily average PM concentration), 0–45 days (45-day moving daily average PM concentration), 0–60 days (60-day moving daily average PM concentration), 0–75 days (75-day moving daily average PM concentration), and 0–90 days (90-day moving daily average PM concentration). When adjusting for the influence of meteorological factors, the corresponding moving average lag structures for the meteorological factors were calculated.

### Statistics

The summary statistics for the characteristics of pregnant women were expressed as frequencies (proportions) for categorical variables and as the means (standard deviation [SD]) or medians (Q1–Q3) for continuous variables. We also conducted univariate logistic regression analysis to evaluate the association between the characteristics of pregnant women and maternal serum 25OHD concentrations during the second trimester. The daily average meteorological variables and daily average PM_2.5_, PM_10_, and 25OHD concentrations were screened for correlations by Pearson’s.

Using generalized estimating equations (GEE), we evaluated the relationships between the cumulative effects of PM on maternal serum 25OHD levels. The cumulative effects of PM were divided into four levels on the basis of quartiles. After adjusting for year and age at blood collection (adjust I) or year, season, and age at blood collection (adjust II), β-values (95% confidence interval [CI]) of the maternal serum 25OHD levels were calculated based on group trends. As part of the sensitivity analysis, we also calculated the β-values (95% CI) for the 25OHD levels when using the concentrations of PM_2.5_ and PM_10_ as continuous variables.

Controlling for the influence of meteorological covariances by applying multivariate linear regression analysis allowed us to evaluate independent associations between the maximum cumulative effects of PM and maternal serum 25OHD. We calculated the results of the unadjusted or minimally adjusted analysis and those from fully adjusted analysis. First, collinearity diagnosis of covariances was performed using variance inflation factor (VIF) analysis (the variable average atmospheric temperature was first removed due to VIF > 10). Then, a judgement on whether to adjust covariances was made using the following principles: Criteria 1, the covariate is added to the basic model or removed from the full model and the matched odds ratio (OR) is changed by at least 10%; Criteria 2: Criteria 1 or a covariate *P*-value of < 0.1 in the univariate model^34^. As part of the sensitivity analysis, we transformed 25OHD quantitative variables into dichotomous qualitative variables (1 = vitamin D deficiency and inadequacy [< 20 ng/mL]; 0 = adequacy [≥ 20 ng/mL]), then the OR and 95% CI for maternal vitamin D deficiency and inadequacy (< 20 ng/mL) associated with a 10 μg/m^3^ increase in PM_2.5_ or PM_10_ was determined.

Non-linear relationships were additionally identified via a generalized additive model (GAM), and on finding a non-linear correlation, the threshold effect in terms of the smoothing curve was calculated using a two-piecewise linear regression model. When a clear ratio was apparent in the smoothing curve, the recursive method was applied to automatically calculate the turning point at which to use the maximum likelihood model^35^. In addition, to test the robustness and potential variation in the different subgroups, we repeated the subgroup analyses while stratifying by season, age, and meteorological factors. The age threshold was derived from the turning point calculated by the GAM followed by an inspection of the modification and interaction of the subgroups with the likelihood ratio test.

All statistical analyses were performed using the Empower Stats (www.empowerstats.com, X&Y solutions, Inc., Boston, MA, USA) and R software version 3.6.3 (http://www.r-project.org). A *P*-value < 0.05 was set as the significance threshold.

### Ethics approval

This study complies with the Declaration of Helsinki and has been approved by the Ethics Committee of the First People’s Hospital of Kunshan (no. 2020-03-046-K01).

## Results

Table 1 displays the data on the pregnant women and their vitamin D statuses. This analysis included 27,768 individuals with a mean age of 28.86 (SD, 4.27) years. The mean (SD) and median (Q1–Q3) values of maternal serum 25OHD concentrations during the second trimester of pregnancy were 17.7 (7.9) ng/mL and 16.0 (12.0–22.0) ng/mL, respectively. Vitamin D deficiency, inadequacy, and adequacy were present in 23.5%, 41.3%, and 35.2% of women, respectively. Univariate logistic regression analysis revealed that maternal 25OHD concentration was positively associated with maternal age and showed seasonal variation, with the peak in September and the nadir in February.

**Table 1:** Characteristics of study participants (N = 27,768).

Characteristics	Statistics	25OHD, mean (SD), ng/mL	β (95% CI)^a^	*P*-value^a^
**Maternal serum 25OHD concentrations during pregnancy continuous, mean (SD)**	17.7 (7.9)			
**Median (Q1–Q3), ng/mL**	16.0 (12.0–22.0)			
**Maternal serum 25OHD concentrations during pregnancy categorical, N (%)**				
Deficiency (< 12 ng/mL)	6532 (23.5%)			
Inadequacy (≥ 12, < 20 ng/mL)	11,465 (41.3%)			
Adequacy (≥ 20 ng/mL)	9771 (35.2%)			
**Age tertile, N (%)**				
Tertile 1 (15–26 years)	8605 (30.99%)	17.18 (7.70)	Reference	–
Tertile 2 (27–29 years)	7948 (28.62%)	17.57 (7.85)	0.39 (0.15, 0.63)	0.001
Tertile 3 (30–47 years)	11,215 (40.39%)	18.08 (8.03)	0.90 (0.68, 1.12)	< 0.0001
*P* for trend				< 0.0001
**Year of blood collection, N (%)**				
2016	5943 (21.40%)	16.28 (7.45)	Reference	–
2017	7410 (26.69%)	18.10 (7.65)	1.82 (1.55, 2.08)	< 0.0001
2018	5616 (20.22%)	17.68 (8.52)	1.40 (1.11, 1.68)	< 0.0001
2019	5204 (18.74%)	18.38 (7.96)	2.10 (1.81, 2.39)	< 0.0001
2020	3595 (12.95%)	17.94 (7.65)	1.65 (1.33, 1.98)	< 0.0001
*P* for trend				< 0.0001
**Month of blood collection, N (%)**				
January	2161 (7.78%)	13.83 (7.02)	Reference	–
February	1549 (5.58%)	13.65 (6.69)	− 0.18 (− 0.66, 0.29)	0.45
March	2285 (8.23%)	14.37 (6.91)	0.54 (0.11, 0.97)	0.01
April	2676 (9.64%)	16.19 (7.24)	2.36 (1.95, 2.77)	< 0.0001
May	2578 (9.28%)	15.76 (6.80)	1.93 (1.52, 2.35)	< 0.0001
June	2505 (9.02%)	18.98 (7.23)	5.15 (4.73, 5.56)	< 0.0001
July	2617 (9.42%)	20.05 (7.39)	6.22 (5.81, 6.64)	< 0.0001
August	2381 (8.57%)	22.10 (7.97)	8.27 (7.85, 8.70)	< 0.0001
September	2276 (8.20%)	22.45 (8.09)	8.62 (8.19, 9.05)	< 0.0001
October	2183 (7.86%)	20.65 (7.78)	6.82 (6.38, 7.25)	< 0.0001
November	2273 (8.19%)	17.53 (7.27)	3.70 (3.27, 4.13)	< 0.0001
December	2284 (8.23%)	14.79 (6.74)	0.96 (0.53, 1.39)	< 0.0001
*P* for trend				< 0.0001
**Season of blood collection, N (%)**				
Spring (March, April and May)	7539 (27.15%)	15.49 (7.03)	Reference	–
Summer (June, July and August)	7503 (27.02%)	20.34 (7.64)	4.85 (4.61, 5.09)	< 0.0001
Autumn (September, October and November)	6732 (24.24%)	20.20 (7.98)	4.71 (4.47, 4.95)	< 0.0001
Winter (December, January and February)	5994 (21.59%)	14.15 (6.85)	− 1.34 (− 1.60, − 1.09)	< 0.0001
*P* for trend				< 0.0001

*25OHD* 25-hydroxy vitamin D, *OR* odds ratio, *CI* confidence interval, *SD* standard deviation.

^a^Crude associations with maternal serum 25OHD concentrations during pregnancy continuous.

Pearson’s correlation analysis was conducted to compare serum 25OHD concentration, meteorological variables, and air pollutant exposure. Figure S2 presents that, except PM_2.5_ vs. sunshine duration (*P*-value = 0.82), all correlations among the variables were statistically significant (*P*-value < 0.001). There was a strong positive correlation between the daily average PM_2.5_ concentration and PM_10_ concentration, and the Pearson coefficient was 0.92. There was also a strong negative correlation between daily average atmospheric temperature and atmospheric pressure, and the Pearson coefficient was − 0.89. There was a moderate negative correlation between daily sunshine duration and relative humidity, and the correlation coefficient was − 0.68.

**Figure 1:**
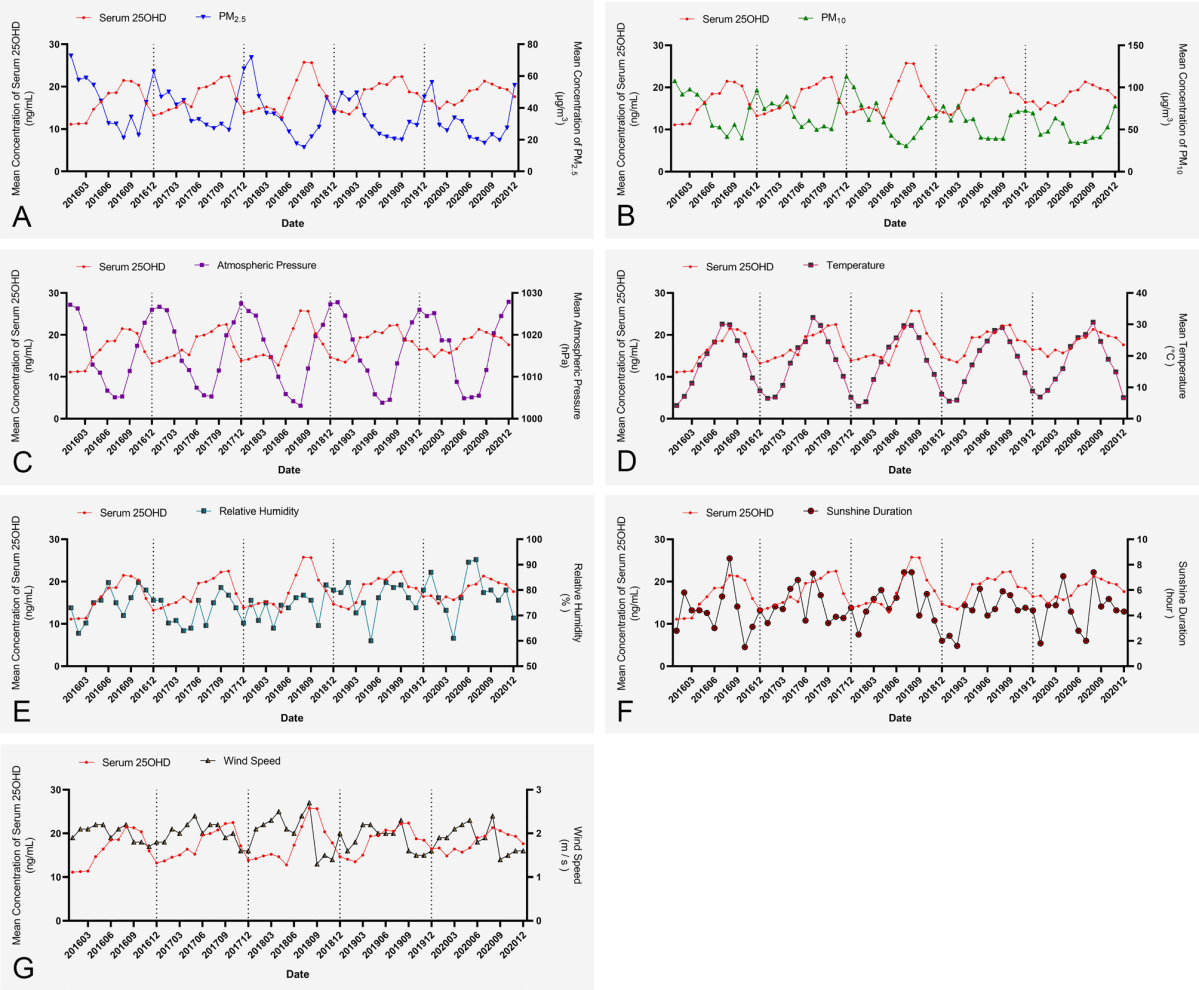
Temporal trends in monthly average serum 25OHD concentration vs. monthly average PM_2.5_ concentration (**A**), PM_10_ concentration (**B**), atmospheric pressure (**C**), temperature (**D**), relative humidity (**E**), sunshine duration (**F**), and wind speed (**G**) from January 2016 to December 2020. *25OHD* 25-hydroxy vitamin D, *PM*_*2.5*_ particulate matter with an aerodynamic diameter of ≤ 2.5 μm, *PM*_*10*_ particulate matter with an aerodynamic diameter of ≤ 10 μm.

Figure 1 shows periodic changes in the above indicators in terms of monthly average values over time. It can be seen that the variation in the average temperature of the last month was most consistent with the periodic variation of monthly average serum 25OHD concentrations. Similar periodic changes were seen for sunshine duration and relative humidity. However, monthly average PM_2.5_ and PM_10_ concentrations and atmospheric pressure showed periodic changes that were diametrically opposite to those of the monthly average serum 25OHD concentration.

**Figure 2:**
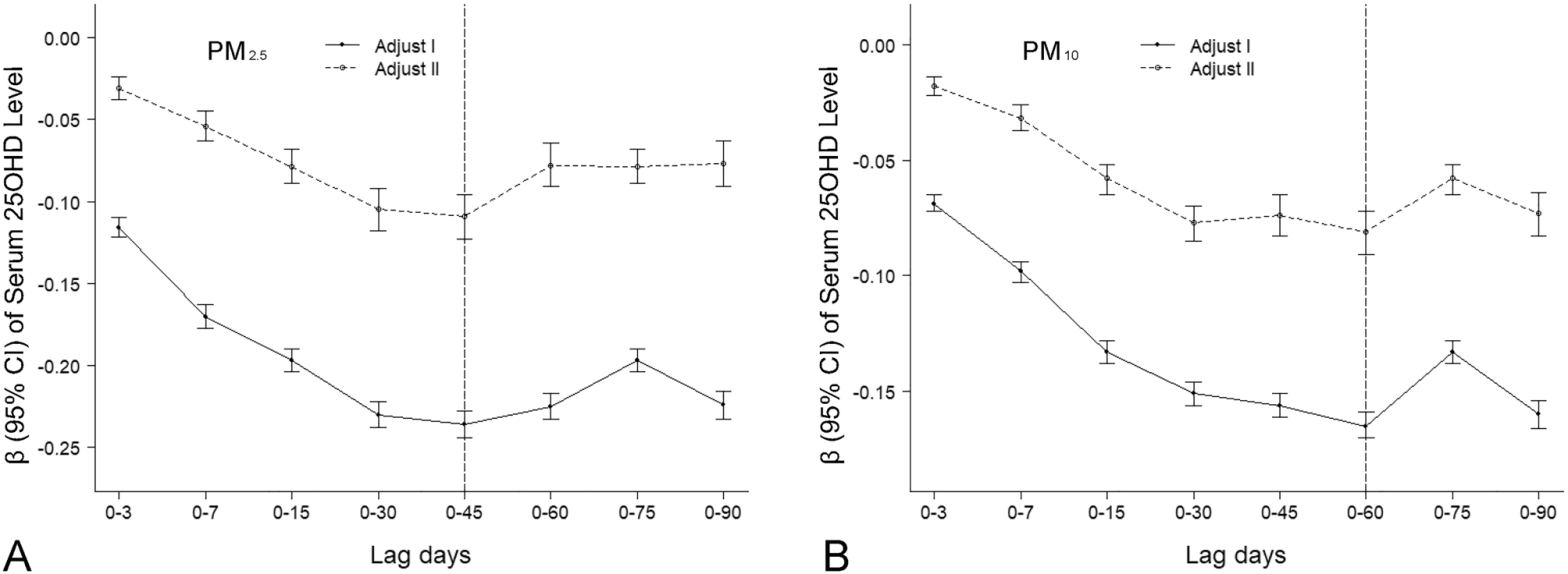
Association among exposure to PM_2.5_ (**A**) or PM_10_ (**B**) and maternal serum 25OHD levels during pregnancy. The cumulative effects of PM were divided into four levels based on quartiles. After adjusting for year and age at blood collection (adjust I) or year, season, and age at blood collection (adjust II), the β-values (95% CI) of maternal serum 25OHD levels during pregnancy were calculated based on the group trend. The dashed vertical line represents lag days corresponding to the maximum cumulative effects. *25OHD* 25-hydroxy vitamin D, *PM*_*2.5*_ particulate matter with an aerodynamic diameter of ≤ 2.5 μm, *PM*_*10*_ particulate matter with an aerodynamic diameter of ≤ 10 μm, *CI* confidence interval.

Figure 2 and Table S1 show the cumulative effects of PM_2.5_ and PM_10_ on maternal serum 25OHD levels during the second trimester of pregnancy. After we adjusted for year, season, and age at blood collection, the maximum cumulative effects occurred at lag 0–45 days of PM_2.5_ and lag 0–60 days of PM_10_. When PM concentrations were used as continues variables, similar results were observed, and these are provided in Table S2.

The independent associations between the maximum cumulative effects of PM and serum 25OHD levels were further investigated, and Table 2 shows the results adjusted for different covariances. In the crude models, the effect sizes of PM_2.5_ (β, − 0.20; 95% CI − 0.21 to − 0.19; *P*-value < 0.00001) and PM_10_ (β, − 0.14; 95% CI − 0.15 to − 0.14; *P*-value < 0.00001) were relatively high. After adjustments for year, season, and age at blood collection (Model 1), the effect sizes of PM_2.5_ (β, − 0.11; 95% CI − 0.13 to − 0.10; *P*-value < 0.00001) and PM_10_ (β, − 0.090; 95% CI − 0.099 to − 0.081; *P*-value < 0.00001) were reduced. On the basis of the Model 1 adjustment, the effect values of PM_2.5_ (β, − 0.042; 95% CI − 0.055 to − 0.028; *P*-value < 0.00001) and PM_10_ (β, − 0.039; 95% CI − 0.049 to − 0.030; *P*-value < 0.00001) were further reduced after adding atmospheric pressure adjustment (Model 2). After further adjustment for sunshine duration, the effect values of Model 3 were similar to those of Model 2. In the fully adjusted Model 4 (adjusted for year, age, season, daily average atmospheric pressure, sunshine duration, relative humidity, and wind speed), there was a negative relationship between the 45-day moving daily average PM_2.5_ concentration and the women’s 25OHD levels (β, − 0.032; 95% CI − 0.046 to − 0.018; *P*-value < 0.00001), and the 60-day moving daily average PM_10_ concentration was negatively associated with 25OHD levels (β, − 0.039; 95% CI − 0.049 to − 0.028; *P*-value < 0.00001). As part of the sensitivity analysis, the crude and adjusted OR for PM exposure’s association with maternal vitamin D deficiency and inadequacy (< 20 ng/mL) were determined, which are given in Table S3. We observed greater odds of maternal vitamin D deficiency and inadequacy (< 20 ng/mL) with higher PM levels. In the fully adjusted Model 4, an increase in the PM_2.5_ 45-day moving daily average by 10 μg/m^3^ was associated with a 12.4% (OR 1.12; 95% CI 1.07 to 1.18, *P*-value < 0.00001) increase in the odds for maternal vitamin D deficiency and inadequacy (< 20 ng/mL). In the fully adjusted Model 4, a 10 μg/m^3^ increase in the PM_10_ 60-day moving daily average was associated with a 11.3% (OR 1.11; 95% CI 1.07 to 1.15, *P*-value < 0.00001) increase in the odds of maternal vitamin D deficiency and inadequacy (< 20 ng/mL).

**Table 2:** Associations of the maximum cumulative effects of PM and maternal serum 25OHD levels during the second trimester in models adjusted for different temporal and meteorological factors.

Model	β (95% CI) *P*-value	
	45-day moving daily average PM_2.5_ concentration	60-day moving daily average PM_10_ concentration
Crude model^a^	− 0.20 (− 0.21, − 0.19) < 0.00001	− 0.14 (− 0.15, − 0.14) < 0.00001
Model 1 (age + year + season)^b^	− 0.11 (− 0.13, − 0.10) < 0.00001	− 0.090 (− 0.099, − 0.081) < 0.00001
Model 2 (model 1 + atmospheric pressure)^c^	− 0.042 (− 0.055, − 0.028) < 0.00001	− 0.039 (− 0.049, − 0.030) < 0.00001
Model 3 (model 2 + sunshine duration)^d^	− 0.039 (− 0.053, − 0.026) < 0.00001	− 0.046 (− 0.055, − 0.036) < 0.00001
Model 4 (model 3 + relative humidity + wind speed)^e^	− 0.032 (− 0.046, − 0.018) < 0.00001	− 0.039 (− 0.049, − 0.028) < 0.00001

*PM* particulate matter, *PM*_*2.5*_ particulate matter with an aerodynamic diameter of ≤ 2.5 μm, *PM*_*10*_ particulate matter with an aerodynamic diameter of ≤ 10 μm, *25OHD* 25-hydroxy vitamin D, *CI* confidence interval.

^a^Not adjusted.

^b^Adjusted for year, age and season.

^c^Adjusted for year, age, season, and corresponding-day moving daily average atmospheric pressure.

^d^Adjusted for year, age, season, the corresponding-day moving daily average atmospheric pressure and sunshine duration.

^e^Adjusted for year, age, season, the corresponding-day moving daily average atmospheric pressure, sunshine duration, relative humidity and wind speed.

**Figure 3:**
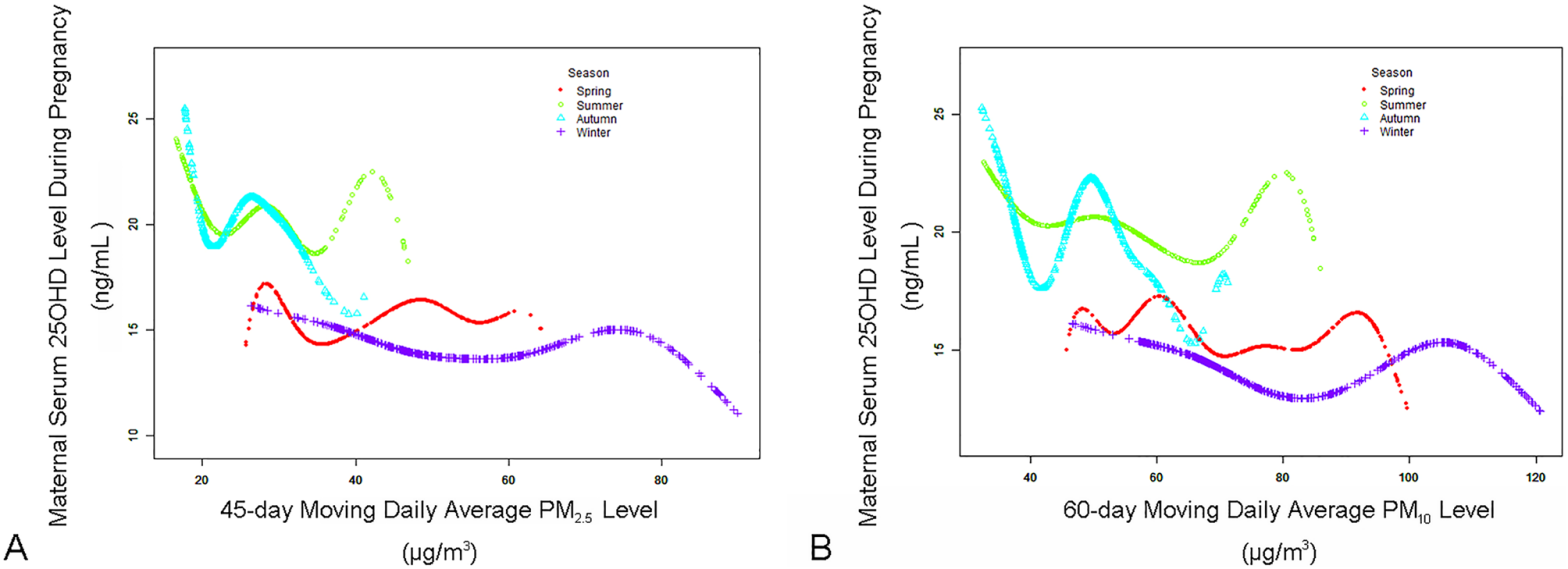
Adjusted smoothed curves for 45-day moving daily average PM_2.5_ concentration (**A**), 60-day moving daily average PM_10_ concentration (**B**), and maternal serum 25OHD levels during pregnancy stratified by seasons. Thresholds were nonlinear associations between PM and 25OHD, as evidenced in generalized additive models. Adjustment factors included year, age, corresponding-day moving daily average atmospheric pressure, sunshine duration, relative humidity, and wind speed. *25OHD* 25-hydroxy vitamin D, *PM*_*2.5*_ particulate matter with an aerodynamic diameter of ≤ 2.5 μm, *PM*_*10*_ particulate matter with an aerodynamic diameter of ≤ 10 μm.

In the subgroup analyses stratified by season, we further investigated the role of season on the association between the maximum cumulative effects of PM and serum 25OHD levels. For PM_2.5_ (Table 3), both linear and nonlinear effect values were higher in summer and autumn and lower in winter and spring (*P*-values for interaction < 0.001). Figure 3A shows the different nonlinear associations between the 45-day moving daily average PM_2.5_ concentration and maternal serum 25OHD levels stratified by season. In addition, we calculated, using the two-piecewise linear regression model, the turning point of the adjusted smoothed curve. Specifically, the difference between the two slopes was at its maximum in autumn, and the turning point (45-day moving daily average PM_2.5_ concentration) was 31.11 μg/m^3^. For PM_10_ (Table 4), linear regression analysis showed a significant interaction for season (interaction *P*-value < 0.001), but the non-linear model showed this interaction was not significant (interaction *P*-value = 0.38). Figure 3B shows the different nonlinear associations between the 60-day moving daily average PM_10_ concentration and maternal serum 25OHD levels stratified by season. It is worth mentioning that, in autumn, the relationship between PM_10_ and 25OHD levels was nonlinear, and the turning point was 38.10 μg/m^3^. Specifically, when the 60-day moving daily average PM_10_ concentration ranged from 32.32 to 38.10 μg/m^3^, a stronger negative relationship was found between PM_10_ and serum 25OHD levels (β, − 1.12; 95% CI − 1.51 to − 0.73; *P*-value < 0.0001; number of pregnant women, 878).

**Table 3:** Threshold effect analysis examining associations between 45-day moving daily average PM_2.5_ levels and maternal serum 25OHD levels during second trimester in subgroups stratified by season of blood collection.

	Spring	Summer	Autumn	Winter	Total
**Model A**^**a**^					*P*-interaction: < 0.001
One line slope, β (95% CI) *P*-value	− 0.027 (− 0.069, 0.014) 0.19	− 0.19 (− 0.25, − 0.13) < 0.0001	− 0.173 (− 0.250, − 0.097) < 0.0001	− 0.029 (− 0.051, − 0.006) 0.01	− 0.032 (− 0.046, − 0.018) < 0.0001
**Model B**^**b**^					*P*-interaction: < 0.001
Turning point (K), μg/m^3^	38.62	35.40	31.11	46.31	20.07
< K, β (95% CI) *P*-value	− 0.23 (− 0.33, − 0.13) < 0.0001	− 0.35 (− 0.43, − 0.27) < 0.0001	0.020 (− 0.086, 0.125) 0.71	− 0.130 (− 0.192, − 0.068) < 0.0001	− 1.80 (− 2.07, − 1.53) < 0.0001
> K, β (95% CI) *P*-value	0.080 (0.016, 0.145) 0.02	0.169 (0.038, 0.300) 0.01	− 0.55 (− 0.71, − 0.39) < 0.0001	− 0.013 (− 0.038, 0.011) 0.28	− 0.022 (− 0.036, − 0.008) 0.002
Slope 2–Slope 1, β (95% CI) *P*-value	0.31 (0.17, 0.45) < 0.0001	0.52 (0.35, 0.69) < 0.0001	− 0.57 (− 0.79, − 0.36) < 0.0001	0.12 (0.05, 0.18) 0.0006	1.78 (1.51, 2.04) < 0.0001
Predicted 25OHD levels at K (95% CI), ng/mL	15.98 (15.65, 16.29)	18.30 (17.98, 18.63)	19.47 (19.14, 19.80)	14.28 (14.02, 14.54)	20.64 (20.49, 20.78)
LRT^c^, *P*-value	< 0.001	< 0.001	< 0.001	< 0.001	< 0.001

*PM*_*2.5*_ particulate matter with an aerodynamic diameter of ≤ 2.5 μm, *25OHD* 25-hydroxy vitamin D, *CI* confidence interval, *LRT* logarithmic likelihood ratio test.

Adjusted for year, age, 45-day moving daily average atmospheric pressure, sunshine duration, relative humidity and wind speed.

^a^Linear analysis, *P*-value < 0.05 indicates a linear relationship.

^b^Non-linear analysis.

^c^*P* < 0.05 means Model B is significantly different from Model A, which indicates a non-linear relationship.

**Table 4:** Threshold effect analysis examining associations between 60-day moving daily average PM_10_ level and maternal serum 25OHD levels during second trimester in subgroups stratified by season of blood collection.

	Spring	Summer	Autumn	Winter	Total
**Model A**^**a**^					*P*-interaction: < 0.001
One line slope, β (95% CI) *P*-value	− 0.10 (− 0.14, − 0.06) < 0.0001	− 0.058 (− 0.098, − 0.019) 0.004	− 0.003 (− 0.062, 0.057) 0.92	− 0.023 (− 0.044, − 0.002) 0.03	− 0.039 (− 0.049, − 0.028) < 0.0001
**Model B**^**b**^					*P*-interaction: 0.38
Turning point (K), μg/m^3^	93.15	45.07	38.10	113.18	38.28
< K, β (95% CI) *P*-value	− 0.080 (− 0.122, − 0.038) 0.0002	− 0.31 (− 0.43, − 0.19) < 0.0001	− 1.12 (− 1.51, − 0.73) < 0.0001	− 0.012 (− 0.034, 0.010) 0.29	− 0.71 (− 0.84, − 0.58) < 0.0001
> K, β (95% CI) *P*-value	− 0.53 (− 0.73, − 0.33) < 0.0001	− 0.030 (− 0.071, 0.012) 0.1648	− 0.046 (− 0.108, 0.015) 0.14	− 0.38 (− 0.58, − 0.18) 0.0002	− 0.038 (− 0.049, − 0.027) < 0.0001
Slope 2–Slope 1, β (95% CI) *P*-value	− 0.45 (− 0.66, − 0.24) < 0.0001	0.28 (0.15, 0.41) < 0.0001	1.07 (0.70, 1.45) < 0.0001	− 0.37 (− 0.57, − 0.17) 0.0004	0.67 (0.54, 0.80) < 0.0001
Predicted 25OHD levels at K (95% CI), ng/mL	14.93 (14.63, 15.23)	20.67 (20.35, 20.99)	20.83 (20.48, 21.18)	12.77 (12.37, 13.17)	20.95 (20.79, 21.11)
LRT^c^, *P*-value	< 0.001	< 0.001	< 0.001	< 0.001	< 0.001

Adjusted for year, age, 60-day moving daily average atmospheric pressure, sunshine duration, relative humidity and wind speed.

*PM*_*10*_ particulate matter with an aerodynamic diameter of ≤ 10 μm, *25OHD* 25-hydroxy vitamin D, *CI* confidence interval, *LRT* logarithmic likelihood ratio test.

^a^Linear analysis, *P*-value < 0.05 indicates a linear relationship.

^b^Non-linear analysis.

^c^*P* < 0.05 means Model B is significantly different from Model A, which indicates a non-linear relationship.

The threshold effect analysis used to examine the associations between maternal age and serum 25OHD levels during the second trimester of pregnancy revealed a stronger positive relationship between age and serum 25OHD levels when the women were 15 to 25 years of age and a weaker positive relationship when they were 25 to 47. The results of four different adjusted models were robust (Fig. S3, Table S4). We then categorized the pregnant women using a threshold of 25 years according to the results of the threshold effect analysis and further investigated the modification effect of age on the association between the maximum cumulative effects of PM and serum 25OHD levels. Table S5 shows that the interaction between age and PM_2.5_ was not significant (linear interaction *P*-value = 0.08 and non-linear interaction *P*-value = 0.06), while Table S6 shows the interaction between age and PM_10_ had a marginally significant effect on serum 25OHD levels (linear *P*-value for interaction = 0.045 and non-linear *P*-value for interaction = 0.04).

In the subgroup analyses stratified by meteorological factors, Tables 5 and 6 showed the associations between PM and serum 25OHD levels during the second trimester of pregnancy were generally modified by meteorological factors, although wind speed had no modification effect on PM_2.5_. Of particular interest, there was a positive association between PM concentration and 25OHD levels under low relative humidity. The result of this stratification was the opposite to the final conclusion.

**Table 5:** Associations between 45-day moving daily average PM_2.5_ levels and maternal serum 25OHD levels during second trimester of pregnancy in subgroups stratified by meteorological factors.

	N	β (95% CI) *P*-value	*P*-value for interaction
**45-day moving daily average atmospheric pressure**			< 0.0001
Tertile 1 (1002.65–1010.69 hPa)	9247	− 0.19 (− 0.25, − 0.13) < 0.0001	
Tertile 2 (1010.74–1020.73 hPa)	9260	− 0.042 (− 0.077, − 0.007) 0.02	
Tertile 3 (1020.75–1028.82 hPa)	9261	− 0.023 (− 0.041, − 0.005) 0.01	
**45-day moving daily average relative humidity**			< 0.0001
Tertile 1 (62.58–71.11%)	9220	0.14 (0.11, 0.18) < 0.0001	
Tertile 2 (71.13–77.04%)	9275	− 0.086 (− 0.111, − 0.060) < 0.0001	
Tertile 3 (77.07–94.33%)	9273	− 0.14 (− 0.17, − 0.10) < 0.0001	
**45-day moving daily average sunshine duration**			< 0.0001
Tertile 1 (1.28–4.10 h)	9136	− 0.044 (− 0.064, − 0.024) < 0.0001	
Tertile 2 (4.12–5.14 h)	9303	− 0.030 (− 0.063, 0.003) 0.07	
Tertile 3 (5.15–8.68 h)	9329	− 0.19 (− 0.24, − 0.14) < 0.0001	
**45-day moving daily average wind speed**			0.24
Tertile 1 (1.36–1.87 m/s)	9138	− 0.068 (− 0.088, − 0.049) < 0.0001	
Tertile 2 (1.88–2.12 m/s)	9117	− 0.040 (− 0.069, − 0.011) 0.007	
Tertile 3 (2.13–2.65 m/s)	9513	− 0.047 (− 0.091, − 0.004) 0.03	

Adjusted for year, season, 45-day moving daily average atmospheric pressure, sunshine duration, relative humidity and wind speed except the subgroup variable.

*PM*_*2.5*_ particulate matter with an aerodynamic diameter of ≤ 2.5 μm, *25OHD* 25-hydroxy vitamin D, *CI* confidence interval.

**Table 6:** Associations between 60-day moving daily average PM_10_ levels and maternal serum 25OHD levels during second trimester of pregnancy in subgroups stratified by meteorological factors.

	N	β (95% CI) *P*-value	*P*-value for interaction
**60-day moving daily average atmospheric pressure**			< 0.0001
Tertile 1 (1003.24–1010.76 hPa)	9241	− 0.15 (− 0.19, − 0.12) < 0.0001	
Tertile 2 (1010.78–1020.92 hPa)	9270	− 0.13 (− 0.16, − 0.11) < 0.0001	
Tertile 3 (1020.97–1028.09 hPa)	9257	− 0.021 (− 0.037, − 0.005) 0.01	
**60-day moving daily average relative humidity**			< 0.0001
Tertile 1 (63.32–71.30%)	9189	0.071 (0.044, 0.099) < 0.0001	
Tertile 2 (71.32–77.18%)	9289	− 0.053 (− 0.071, − 0.035) < 0.0001	
Tertile 3 (77.23–92.27%)	9290	− 0.074 (− 0.096, − 0.051) < 0.0001	
**60-day moving daily average sunshine duration**			< 0.0001
Tertile 1 (1.84–4.07 h)	9237	− 0.026 (− 0.043, − 0.008) 0.003	
Tertile 2 (4.08–5.15 h)	9234	− 0.057 (− 0.078, − 0.035) < 0.0001	
Tertile 3 ( 5.16–7.90 h)	9297	− 0.128 (− 0.157, − 0.099) < 0.0001	
**60-day moving daily average wind speed**			0.003
Tertile 1 (1.34–1.89 m/s)	8993	− 0.043 (− 0.059, − 0.027) < 0.0001	
Tertile 2 (1.90–2.11 m/s)	9306	− 0.078 (− 0.102, − 0.054) < 0.0001	
Tertile 3 (2.12–2.58 m/s)	9469	− 0.094 (− 0.124, − 0.065) < 0.0001	

Adjusted for year, season, 60-day moving daily average atmospheric pressure, sunshine duration, relative humidity and wind speed except the subgroup variable.

*PM*_*10*_ particulate matter with an aerodynamic diameter of ≤ 10 μm, *25OHD* 25-hydroxy vitamin D, *CI* confidence interval.

## Discussion

Based on our knowledge of the literature, this epidemiological study is the first to focus on the independent relationship between PM and maternal serum 25OHD levels during the second trimester of pregnancy after adjusting for meteorological factors. We found vitamin D deficiency, inadequacy, and adequacy in 23.5%, 41.3%, and 35.2% of pregnant women during the second trimester of pregnancy, respectively. We found that the maximum cumulative effects of PM_2.5_ occurred at lag 45 days and the maximum cumulative effects of PM_10_ occurred at lag 60 days. However, the effect values were drastically reduced after adjusting for temporal and/or meteorological factors. The results indicated that PM has a limited influence on maternal serum 25OHD levels.

The list of studies that have linked VDD to complications of pregnancy continues to grow: vitamin D status has been associated with gestational diabetes^36–39^, aeroallergen sensitization^40^, and markers of regulatory immunity^41^. A meta-analysis of eight studies found a significant association between VDD and the risk of pre-eclampsia, which was more evident in studies that defined VDD as 25OHD < 50 nmol/L and those from the USA^42^. In addition, a meta-analysis of 24 observational studies confirmed the association between VDD (< 50 nmol/L) and an increased risk of preterm birth (OR 1.58; 95% CI 1.08 to 2.31)^43^. With respect to birthweight, a meta-analysis of three observational studies found a weak positive association between maternal vitamin D status and birthweight after adjusting for potential confounders^44^. In addition, recent reviews suggested that appropriate levels of vitamin D during pregnancy are associated with less morbidity during pregnancy^45,46^.

Zhao et al. reported an association between prenatal exposure to higher PM_2.5_ and PM_10_ levels and a decrease in circulating 25OHD concentrations in women in the third trimester and the entire pregnancy^24^. They reported a 10 μg/m^3^ increase in PM_2.5_ and PM_10_ exposure during the entire pregnancy was associated with a 4.62% (95% CI 26.31% to 22.93%) and 5.06% (95% CI 26.50% to 23.62%) decrease in 25OHD levels, respectively^24^. In contrast to the analysis they conducted, we first investigated the time window for the largest cumulative effect of PM at the individual level and then adjusted for temporal and meteorological factors. Although both effect sizes of PM_2.5_ and PM_10_ in the fully adjusted models were small (β-value of PM_2.5_ =  − 0.032; β-value of PM_10_ =  − 0.039, respectively), they were highly significant (*P*-value < 0.00001). The β-values showed two large drops, one after adjusting for season and the other after adjusting for atmospheric pressure. Due to the collinearity of atmospheric pressure and temperature, it can be inferred that season and atmospheric pressure/temperature may have been important confounders in the regression model. Thus, in some correlation analyses of PM and vitamin D levels, the independent role of PM may have been overestimated when there was no adjustment for meteorological factors. On the other hand, there is now evidence that 25OHD accumulates in skeletal muscle cells, which provide a functional store during the winter months^47,48^. The mechanism is mediated by the muscle cell uptake of circulating vitamin-D-binding protein (DBP) through a megalin–cubilin membrane transport process^47^. If ways to optimize the efficiency of the muscle conservation mechanism for 25OHD could be found, e.g., a pharmacological agent or some exercise regime, perhaps that optimization would ensure that vitamin D status is also optimized by a process that has evolved to adapt to seasonal changes in vitamin D supply^48^. Therefore, we also proposed, when PM levels are high in winter and spring (when the population’s vitamin D levels are also low), pregnant women, besides taking vitamin D supplements, should keep participating in outdoor activities while taking PM protection measures, e.g., wearing a facemask or face covering and avoiding high-PM areas such as alongside high-volume traffic roads, to increase their vitamin D levels.

Indeed, in areas with distinct seasons, 25OHD concentrations in the population fluctuate over time^29,49–52^. Many studies showed season to be the primary factor affecting serum 25OHD levels^15,29,53–55^. Periodic changes in the seasons affect periodic changes in meteorological factors, such as temperature, humidity, and sunshine duration. Hence, unlike other studies that only adjusted for season, our study precisely adjusted for meteorological factor exposure at the individual level.

On the other hand, meteorological factors are also related to periodic changes in PM concentrations. Weather and climate are the most influential forces affecting the chemistry and atmospheric residence time of PM^56^. In a study in China, the geographical and temporal variations in PM levels and coinciding meteorological conditions were analyzed for 366 cities, and peak PM concentrations occurred in winter in most regions, and there were negative correlations between PM levels and precipitation, relative humidity, air temperature, and wind speed but a positively correlation was found between PM levels and surface pressure^57^. Guan et al.^58^ reported the spatiotemporal variability of PM in three cities in western China and the influence of meteorological factors on PM. Crawford et al.^59^, who analyzed the impact of meteorology on particulate source types in Lucas Heights, Australia, reported that temperature significantly affected PM_2.5_ levels. An Indian study reported that PM displayed substantial seasonal variations and a strong negative association with temperature, with considerable dependency levels^56^.

Over 90% of the vitamin D in the body is synthesized in human skin after the exposer of precursor 7-dehydrocholesterol to UVB (290–315 nm) radiation from the sun^60^. The strength of the UVB during sun exposure, and therefore the amount of vitamin D synthesized, is affected by solar zenith angle (SZA)—the angle between the local vertical and the sun’s position. Hence, the most intense solar radiation occurs when the SZA is small, i.e., which at lower altitudes, is around 11:00–15:00 h in the summer, at which time the synthesis of vitamin D by the skin is most active^61^. At latitudes > 50°, human skin participates in very little vitamin D synthesis during winter and spring for all skin types and ethnicities^62^. In addition, the amount of vitamin D produced is dependent on local weather conditions, such as the percentage cloud cover, which filters UVB radiation, and has an impact in all seasons and hours of the day^50^. Thus, because PM can absorb and diffuse solar irradiation, some scholars have hypothesized that PM can indirectly reduce vitamin D formation by reducing UVB exposure^23,24^. However, other scholars have argued that there is significant spatiotemporal variation in the morphology, chemical makeup, density of PM, and it is difficult to determine its effect on UV radiation^63^. More recently, PM_10_ was found to be a significant negative predictor of solar UVB radiation, but the effect of PM_10_ was miniscule^27^. This conclusion can be used to further deduce that PM_10_ has an effect on vitamin D levels, but the effect is small, which is consist with the results of our study. Combined with the above views, we propose a hypothesis (see Fig. S4) that PM and meteorological factors indirectly influence the cyclical changes of vitamin D in pregnant women by impacting the level of personal UVB exposure. For example, in winter, when the temperature is lower and there are fewer hours of sunshine, the PM concentration is higher, but pregnant women spend less time outdoors and wear more clothes, which lead to lower solar UVB radiation exposure, reducing the synthesis of vitamin D.

In the subgroup analysis, with the exception of the low relative humidity, a negative effect between PM and 25OHD was evident in all subgroups considered and after careful adjustments. In the interaction analysis, season and most meteorological factors interacted with the association between PM and 25OHD. However, the mechanisms of their interactions were unclear and need to be further investigated in the future.

There were some limitations regarding our study. First, our results were obtained from a Chinese population of pregnant women during the second trimester and cannot be extrapolated to other populations. Second, the study was an analytical retrospective study and hence provides limited evidence that PM exposure and vitamin D outcomes were related, and the difference between cause and effect is uncertain. Third, demographic information was lacking (education level, vitamin D/calcium supplement use, outdoor activities, use of sun protection, and BMI, etc.) for the individuals whose test results were used. However, valuable insights can be gleaned from the study, as it involved the retrospective analysis of a large dataset from a prenatal examination population. We used precise adjustments at the individual level to reveal whether PM levels are independently associated with 25OHD concentration. In the future, a more informative vitamin D database will be established, and vitamin D supplementation will be studied. Fourth, solar UVB dose information was not studied; thus, this important indicator could be included in the future studies.

## Conclusions

Our study showed there was a small, independent, negative association between PM in the atmosphere and maternal serum 25OHD levels during the second trimester of pregnancy after adjusting for temporal and/or meteorological factors, which indicates that PM may have a limited influence on maternal serum 25OHD levels. Besides taking vitamin D supplements, pregnant women should keep participating in outdoor activities while taking PM protection measures to improve their vitamin D levels when PM levels are high in winter and spring.

## Supplementary Information

Supplementary Figure S1.

Supplementary Figure S2.

Supplementary Figure S3.

Supplementary Figure S4.

Supplementary Information.

Supplementary Table S1.

Supplementary Table S2.

Supplementary Table S3.

Supplementary Table S4.

Supplementary Table S5.

Supplementary Table S6.

## Data Availability

All data generated or analysed during this study are included in this published article and its Supplementary Information files.
