# Supplementary figures and images for: Particulate matter may have a limited influence on maternal vitamin D levels
Source: Sci Rep. 2022 Oct 7;12:16807. doi: 10.1038/s41598-022-21383-1 (PMC9546910; doi:10.1038/s41598-022-21383-1)

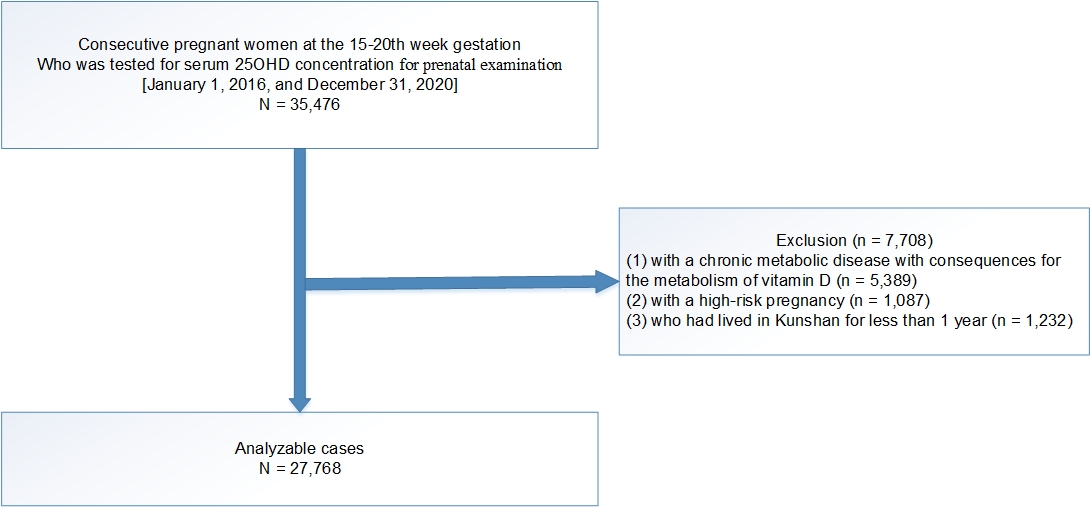


**Figure S1.** Flow chart for this study. 25OHD, 25-hydroxy vitamin D.

Supplement: Supplementary file 1 — Supplementary Figure S1. [file 41598_2022_21383_MOESM1_ESM.docx]
