# Supplementary material for: Particulate matter may have a limited influence on maternal vitamin D levels
Source: Sci Rep. 2022 Oct 7;12:16807. doi: 10.1038/s41598-022-21383-1 (PMC9546910; doi:10.1038/s41598-022-21383-1)
Supplement: Supplementary file 2 — Supplementary Figure S2. [file 41598_2022_21383_MOESM2_ESM.docx]

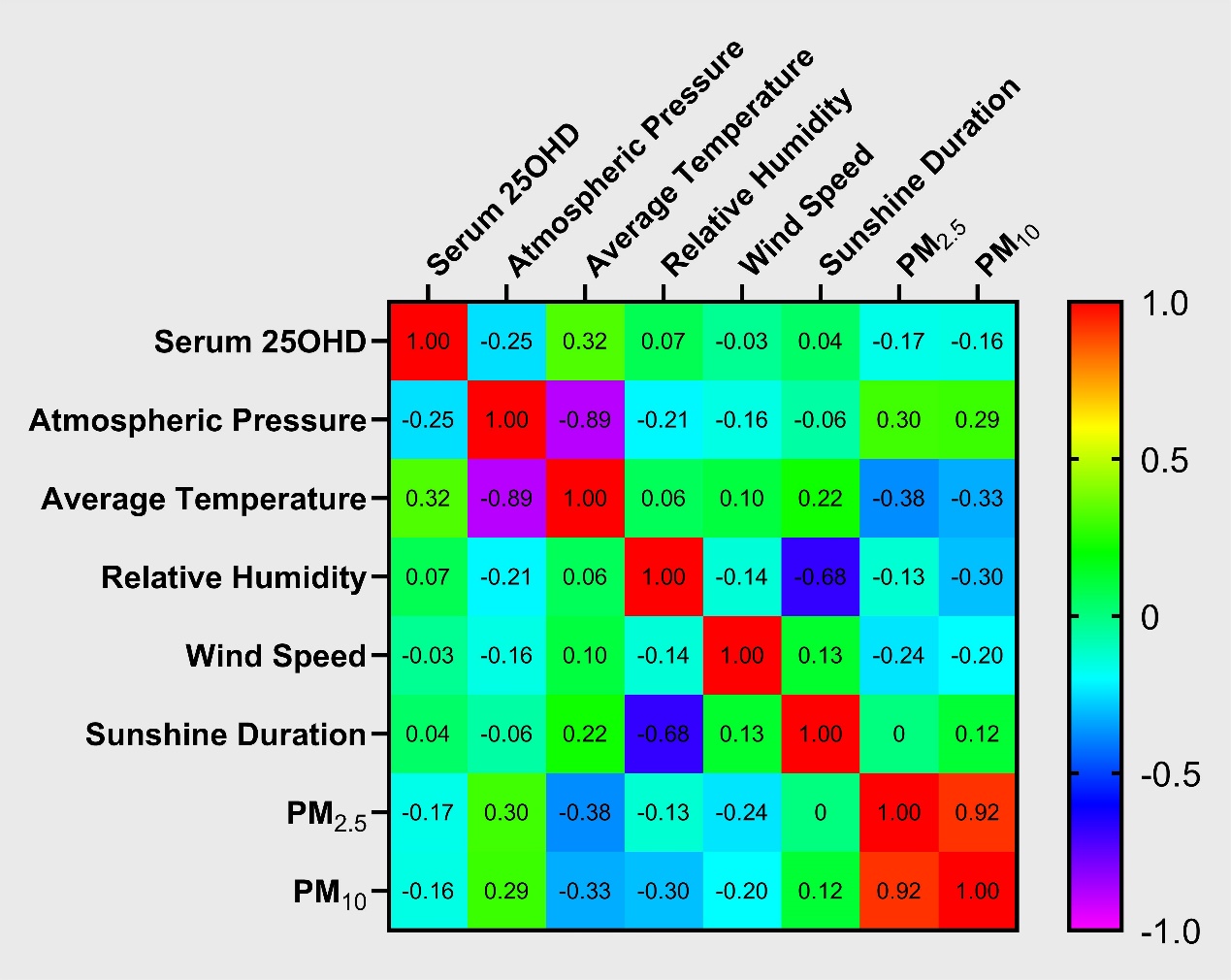


**Figure S2**. Heat map graphic showing positive and negative correlations among maternal serum 25OHD concentrations during pregnancy, meteorological variables (daily average atmospheric temperature [℃], atmospheric pressure [hPa], relative humidity [%], sunshine duration [hour], and wind speed [m/s]), and air pollutant exposure (daily average PM_2.5_ concentration [μg/m^3^] and PM_10_ concentration [μg/m^3^]). Correlation coefficients are listed in each small square. With the exception of PM_2.5_ vs. sunshine duration (*P*-value = 0.82), correlations between the variables were statistically significant (*P*-value < 0.001). 25OHD, 25-hydroxy vitamin D; PM_2.5_, particulate matter with an aerodynamic diameter of ≤2.5 μm; PM_10_, particulate matter with an aerodynamic diameter of ≤10 μm.
