# Supplementary material for: Particulate matter may have a limited influence on maternal vitamin D levels
Source: Sci Rep. 2022 Oct 7;12:16807. doi: 10.1038/s41598-022-21383-1 (PMC9546910; doi:10.1038/s41598-022-21383-1)
Supplement: Supplementary file 3 — Supplementary Figure S3. [file 41598_2022_21383_MOESM3_ESM.docx]

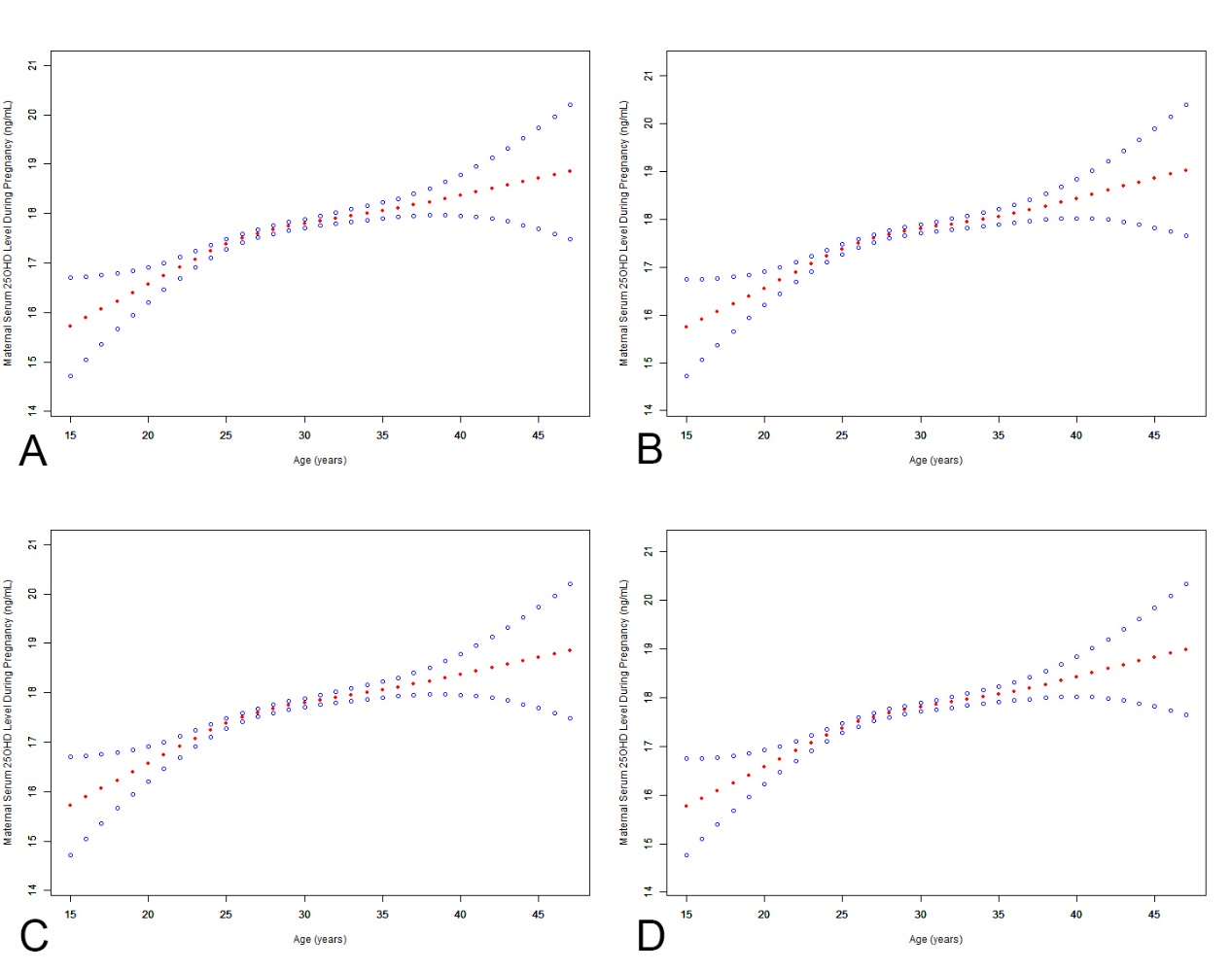


**Figure S3.** Adjusted smoothed curves of maternal age and serum 25OHD levels. A, adjusted for year and 45-day moving daily average PM_2.5_ level; B, adjusted for year, season, 45-day moving daily average PM_2.5_ level, daily average atmospheric pressure, sunshine duration, relative humidity, and wind speed; C, adjusted for year and 60-day moving daily average PM_10_ level; D, adjusted for year, season, 60-day moving daily average PM_10_ level, daily average atmospheric pressure, sunshine duration, relative humidity, and wind speed. There was a threshold nonlinear association between maternal age and serum 25OHD levels (red dotted line), as evidenced by the generalized additive model. The area between two blue dotted lines was expressed as a 95% CI. 25OHD, 25-hydroxy vitamin D; PM_2.5_, particulate matter with an aerodynamic diameter of ≤2.5 μm; PM_10_, particulate matter with an aerodynamic diameter of ≤10 μm; CI, confidence interval.
