# Supplementary material for: Particulate matter may have a limited influence on maternal vitamin D levels
Source: Sci Rep. 2022 Oct 7;12:16807. doi: 10.1038/s41598-022-21383-1 (PMC9546910; doi:10.1038/s41598-022-21383-1)
Supplement: Supplementary file 4 — Supplementary Figure S4. [file 41598_2022_21383_MOESM4_ESM.docx]

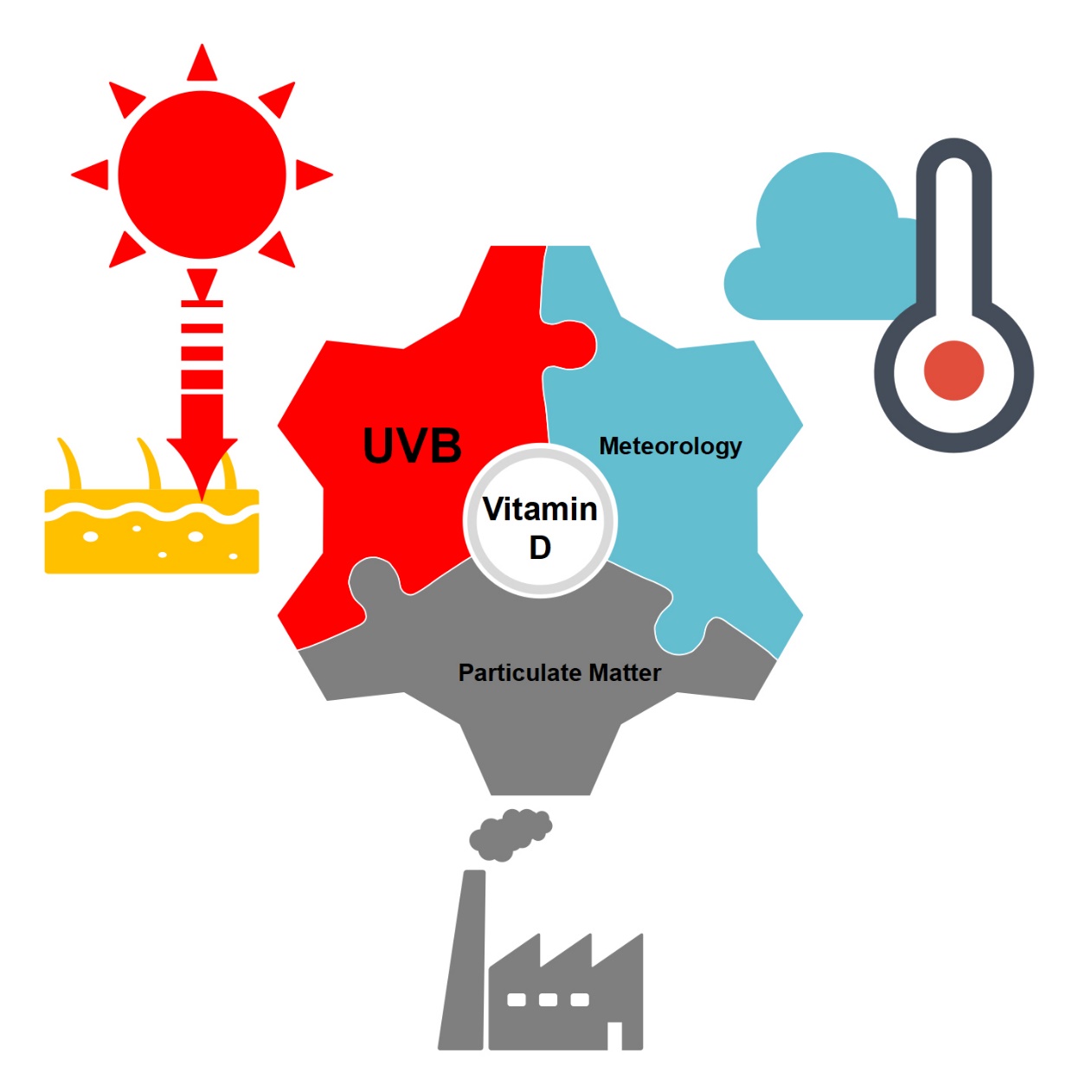


**Figure S4.** A hypothesis can be proposed, that, combined with meteorological factors, high levels of PM reduce solar UVB radiation exposure and hence indirectly reduce the synthesis of vitamin D. PM, particulate matter; UV, ultraviolet.
