# Supplementary material for: Particulate matter may have a limited influence on maternal vitamin D levels
Source: Sci Rep. 2022 Oct 7;12:16807. doi: 10.1038/s41598-022-21383-1 (PMC9546910; doi:10.1038/s41598-022-21383-1)
Supplement: Supplementary file 6 — Supplementary Table S1. [file 41598_2022_21383_MOESM6_ESM.docx]

Table S1. Associations of cumulative effects^a^ of PM (quartiles) and maternal serum 25OHD levels during second trimester

| Exposure | Adjust I^b^ | | Adjust II^c^ | |
| --- | --- | --- | --- | --- |
|  | β (95% CI) | *P*-value | β (95% CI) | *P*-value |
| PM_2.5_ lag days, d |  |  |  |  |
| 0-3 | -0.12 (-0.12, -0.11) | <0.00001 | -0.031 (-0.038, -0.024) | <0.00001 |
| 0-7 | -0.17 (-0.18, -0.16) | <0.00001 | -0.054 (-0.063, -0.045) | <0.00001 |
| 0-15 | -0.20 (-0.20, -0.19) | <0.00001 | -0.079 (-0.089, -0.068) | <0.00001 |
| 0-30 | -0.23 (-0.24, -0.22) | <0.00001 | -0.105 (-0.118, -0.092) | <0.00001 |
| 0-45 | **-0.24 (-0.24, -0.23)** | **<0.00001** | **-0.109 (-0.123, -0.096)** | **<0.00001** |
| 0-60 | -0.23 (-0.23, -0.22) | <0.00001 | -0.078 (-0.091, -0.064) | <0.00001 |
| 0-75 | -0.20 (-0.20, -0.19) | <0.00001 | -0.079 (-0.089, -0.068) | <0.00001 |
| 0-90 | -0.22 (-0.23, -0.22) | <0.00001 | -0.077 (-0.091, -0.063) | <0.00001 |
| PM_10_ lag days, d |  |  |  |  |
| 0-3 | -0.069 (-0.072, -0.065) | <0.00001 | -0.018 (-0.022, -0.014) | <0.00001 |
| 0-7 | -0.098 (-0.103, -0.094) | <0.00001 | -0.032 (-0.037, -0.026) | <0.00001 |
| 0-15 | -0.13 (-0.14, -0.13) | <0.00001 | -0.058 (-0.065, -0.052) | <0.00001 |
| 0-30 | -0.15 (-0.16, -0.15) | <0.00001 | -0.077 (-0.085, -0.070) | <0.00001 |
| 0-45 | -0.16 (-0.16, -0.15) | <0.00001 | -0.074 (-0.083, -0.065) | <0.00001 |
| 0-60 | **-0.17 (-0.17, -0.16)** | **<0.00001** | **-0.081 (-0.091, -0.072)** | **<0.00001** |
| 0-75 | -0.13 (-0.14, -0.13) | <0.00001 | -0.058 (-0.065, -0.052) | <0.00001 |
| 0-90 | -0.16 (-0.17, -0.15) | <0.00001 | -0.073 (-0.083, -0.064) | <0.00001 |

^a^Cumulative effects of PM_2.5_/PM_10_ were divided into four levels based on quartiles, and estimates were calculated based on group trends.

^b^Adjusted for year and age.

^c^Adjusted for year, age and season.

Abbreviations: PM, particulate matter; PM_2.5_, particulate matter with an aerodynamic diameter of ≤2.5 μm; PM_10_, particulate matter with an aerodynamic diameter of ≤10 μm; 25OHD, 25-hydroxy vitamin D; CI, confidence interval.
