# Supplementary material for: Particulate matter may have a limited influence on maternal vitamin D levels
Source: Sci Rep. 2022 Oct 7;12:16807. doi: 10.1038/s41598-022-21383-1 (PMC9546910; doi:10.1038/s41598-022-21383-1)
Supplement: Supplementary file 7 — Supplementary Table S2. [file 41598_2022_21383_MOESM7_ESM.docx]

Table S2. Associations of the cumulative effects^a^ of PM (continuous) and maternal serum 25OHD levels during the second trimester

| Exposure | Adjust I^b^ | | Adjust II^c^ | |
| --- | --- | --- | --- | --- |
|  | β (95% CI) | *P*-value | β (95% CI) | *P*-value |
| PM_2.5_ lag days, d |  |  |  |  |
| 0-3 | -0.077 (-0.082, -0.073) | <0.00001 | -0.018 (-0.023, -0.013) | <0.00001 |
| 0-7 | -0.12 (-0.13, -0.12) | <0.00001 | -0.037 (-0.044, -0.031) | <0.00001 |
| 0-15 | -0.16 (-0.17, -0.16) | <0.00001 | -0.068 (-0.077, -0.059) | <0.00001 |
| 0-30 | -0.20 (-0.20, -0.19) | <0.00001 | -0.103 (-0.114, -0.092) | <0.00001 |
| 0-45 | -0.21 (-0.22, -0.20) | <0.00001 | -0.11 (-0.13, -0.10) | <0.00001 |
| 0-60 | -0.22 (-0.22, -0.21) | <0.00001 | -0.108 (-0.120, -0.096) | <0.00001 |
| 0-75 | -0.16 (-0.17, -0.16) | <0.00001 | -0.068 (-0.077, -0.059) | <0.00001 |
| 0-90 | -0.22 (-0.22, -0.21) | <0.00001 | -0.104 (-0.117, -0.092) | <0.00001 |
| PM_10_ lag days, d |  |  |  |  |
| 0-3 | -0.052 (-0.055, -0.049) | <0.00001 | -0.012 (-0.016, -0.009) | <0.00001 |
| 0-7 | -0.082 (-0.086, -0.079) | <0.00001 | -0.026 (-0.030, -0.021) | <0.00001 |
| 0-15 | -0.12 (-0.12, -0.11) | <0.00001 | -0.051 (-0.057, -0.046) | <0.00001 |
| 0-30 | -0.14 (-0.15, -0.14) | <0.00001 | -0.078 (-0.085, -0.071) | <0.00001 |
| 0-45 | -0.15 (-0.16, -0.15) | <0.00001 | -0.088 (-0.097, -0.080) | <0.00001 |
| 0-60 | -0.16 (-0.16, -0.15) | <0.00001 | -0.090 (-0.099, -0.081) | <0.00001 |
| 0-75 | -0.12 (-0.12, -0.11) | <0.00001 | -0.051 (-0.057, -0.046) | <0.00001 |
| 0-90 | -0.15 (-0.16, -0.15) | <0.00001 | -0.083 (-0.092, -0.074) | <0.00001 |

^a^Cumulative effects of PM were calculated as a continuous variable.

^b^Adjusted for year and age.

^c^Adjusted for year, age and season.

Abbreviations: PM, particulate matter; PM_2.5_, particulate matter with an aerodynamic diameter of ≤2.5 μm; PM_10_, particulate matter with an aerodynamic diameter of ≤10 μm; 25OHD, 25-hydroxy vitamin D; CI, confidence interval.
