# Supplementary material for: Particulate matter may have a limited influence on maternal vitamin D levels
Source: Sci Rep. 2022 Oct 7;12:16807. doi: 10.1038/s41598-022-21383-1 (PMC9546910; doi:10.1038/s41598-022-21383-1)
Supplement: Supplementary file 8 — Supplementary Table S3. [file 41598_2022_21383_MOESM8_ESM.docx]

Table S3. Associations between particulate matter exposure and risk of maternal vitamin D deficiency and inadequacy (<20 ng/mL)

| Model | OR^f^ (95% CI)  *P*-value | |
| --- | --- | --- |
|  | 45-day moving daily average PM_2.5_ concentration | 60-day moving daily average PM_10_ concentration |
| Crude Model^a^ | 1.66 (1.62, 1.70) <0.00001 | 1.40 (1.38, 1.42) <0.00001 |
| Model 1 (Age + Year + Season)^b^ | 1.39 (1.33, 1.45) <0.00001 | 1.26 (1.23, 1.30) <0.00001 |
| Model 2 (Model 1 + Atmospheric Pressure)^c^ | 1.14 (1.09, 1.20) <0.00001 | 1.10 (1.07, 1.14) <0.00001 |
| Model 3 (Model 2 + Sunshine Duration)^d^ | 1.14 (1.08, 1.19) <0.00001 | 1.12 (1.09, 1.16) <0.00001 |
| Model 4 (Model 3 + Relative Humidity +Wind Speed)^e^ | 1.12 (1.07, 1.18) <0.00001 | 1.11 (1.07, 1.15) <0.00001 |

^a^Not adjusted.

^b^Adjusted for year, age and season.

^c^Adjusted for year, age, season and the corresponding-day moving daily average atmospheric pressure.

^d^Adjusted for year, age, season, the corresponding-day moving daily average atmospheric pressure and sunshine duration.

^e^Adjusted for year, age, season, the corresponding-day moving daily average atmospheric pressure, sunshine duration, relative humidity and wind speed.

^f^OR and 95% CI was calculated on basis of a 10 μg/m^3^ increase in PM_2.5_ or PM_10_ exposure.

Abbreviations: PM, particulate matter; PM_2.5_, particulate matter with an aerodynamic diameter of ≤2.5 μm; PM_10_, particulate matter with an aerodynamic diameter of ≤10 μm; 25-hydroxy vitamin D; OR, odds ratio; CI, confidence interval.
