# Supplementary material for: Particulate matter may have a limited influence on maternal vitamin D levels
Source: Sci Rep. 2022 Oct 7;12:16807. doi: 10.1038/s41598-022-21383-1 (PMC9546910; doi:10.1038/s41598-022-21383-1)
Supplement: Supplementary file 9 — Supplementary Table S4. [file 41598_2022_21383_MOESM9_ESM.docx]

Table S4. Threshold effect analysis examining associations between maternal age and serum 25OHD levels during second trimester in different models

|  | Model I^d^ | Model II^e^ | Model III^f^ | Model IV^g^ |
| --- | --- | --- | --- | --- |
| Model A^a^ |  |  |  |  |
| One line slope, β (95%CI) *P*-value | 0.08 (0.06, 0.10) <0.0001 | 0.08 (0.06, 0.10) <0.0001 | 0.08 (0.06, 0.10) <0.0001 | 0.08 (0.06, 0.10) <0.0001 |
| Model B^b^ |  |  |  |  |
| Turning point (K), years | 25 | 25 | 25 | 25 |
| < K, β (95%CI) *P*-value | 0.23 (0.13, 0.32) <0.0001 | 0.22 (0.13, 0.31) <0.0001 | 0.21 (0.12, 0.31) <0.0001 | 0.22 (0.13, 0.31) <0.0001 |
| > K, β (95%CI) *P*-value | 0.06 (0.03, 0.08) <0.0001 | 0.06 (0.03, 0.08) <0.0001 | 0.06 (0.03, 0.08) <0.0001 | 0.06 (0.04, 0.08) <0.0001 |
| Slope 2 – Slope 1, β (95%CI) *P*-value | -0.17 (-0.27, -0.06) 0.002 | -0.16 (-0.26, -0.06) 0.002 | -0.16 (-0.26, -0.05) 0.004 | -0.16 (-0.26, -0.06) 0.002 |
| Predicted 25OHD levels at K (95% CI), ng/mL | 17.36 (17.21, 17.52) | 17.36 (17.21, 17.52) | 17.36 (17.21, 17.52) | 17.36 (17.21, 17.52) |
| LRT^c^, *P*-value | 0.002 | 0.002 | 0.004 | 0.002 |

^a^Linear analysis, *P*-value < 0.05 indicates a linear relationship.

^b^Non-linear analysis.

^c^*P* < 0.05 means Model B is significantly different from Model A, which indicates a non-linear relationship.

^d^Adjusted for year and 45-day moving daily average PM_2.5_ level.

^e^Adjusted for year, season, 45-day moving daily average PM_2.5_ level, daily average atmospheric pressure, sunshine duration, relative humidity and wind speed.

^f^Adjusted for year and 60-day moving daily average PM_10_ level.

^g^Adjusted for year, season, 60-day moving daily average PM_10_ level, daily average atmospheric pressure, sunshine duration, relative humidity and wind speed.

Abbreviations: PM_2.5_, particulate matter with an aerodynamic diameter of ≤2.5 μm; PM_10_, particulate matter with an aerodynamic diameter of ≤10 μm; 25OHD, 25-hydroxy vitamin D; CI, confidence interval; LRT, logarithmic likelihood ratio test.
