# Supplementary material for: Particulate matter may have a limited influence on maternal vitamin D levels
Source: Sci Rep. 2022 Oct 7;12:16807. doi: 10.1038/s41598-022-21383-1 (PMC9546910; doi:10.1038/s41598-022-21383-1)
Supplement: Supplementary file 10 — Supplementary Table S5. [file 41598_2022_21383_MOESM10_ESM.docx]

Table S5. Threshold effect analysis examining associations between 45-day moving daily average PM_2.5_ levels and maternal serum 25OHD levels during second trimester in subgroups stratified by age

|  | Age ≤25 | Age >25 | Total |
| --- | --- | --- | --- |
| Model A^a^ |  |  | *P*-interaction: 0.08 |
| One line slope, β (95%CI) *P*-value | -0.038 (-0.067, -0.009) 0.01 | -0.032 (-0.048, -0.016) <0.0001 | -0.033 (-0.047, -0.019) <0.0001 |
| Model B^b^ |  |  | *P*-interaction: 0.06 |
| Turning point (K), μg/m^3^ | 19.98 | 20.11 | 20.07 |
| < K, β (95%CI) *P*-value | -1.54 (-2.10, -0.97) <0.0001 | -1.86 (-2.17, -1.56) <0.0001 | -1.79 (-2.06, -1.53) <0.0001 |
| > K, β (95%CI) *P*-value | -0.031 (-0.060, -0.002) 0.04 | -0.021 (-0.037, -0.005) 0.01 | -0.023 (-0.037, -0.009) 0.001 |
| Slope 2 – Slope 1, β (95%CI) *P*-value | 1.51 (0.94, 2.07) <0.0001 | 1.84 (1.54, 2.14) <0.0001 | 1.77 (1.50, 2.04) <0.0001 |
| Predicted 25OHD levels at K (95% CI), ng/mL | 19.94 (19.64, 20.25) | 20.81 (20.64, 20.98) | 20.64 (20.49, 20.78) |
| LRT^c^, *P*-value | <0.001 | <0.001 | <0.001 |

Adjusted for year, season, 45-day moving daily average atmospheric pressure, sunshine duration, relative humidity and wind speed.

^a^Linear analysis, *P*-value <0.05 indicates a linear relationship.

^b^Non-linear analysis.

^c^*P* < 0.05 means Model B is significantly different from Model A, which indicates a non-linear relationship.

Abbreviations: PM_2.5_, particulate matter with an aerodynamic diameter of ≤2.5 μm; 25OHD, 25-hydroxy vitamin D; CI, confidence interval; LRT, logarithmic likelihood ratio test.
