# Supplementary material for: Particulate matter may have a limited influence on maternal vitamin D levels
Source: Sci Rep. 2022 Oct 7;12:16807. doi: 10.1038/s41598-022-21383-1 (PMC9546910; doi:10.1038/s41598-022-21383-1)
Supplement: Supplementary file 11 — Supplementary Table S6. [file 41598_2022_21383_MOESM11_ESM.docx]

Table S6. Threshold effect analysis examining associations between 60-day moving daily average PM_10_ levels and maternal serum 25OHD levels during second trimester in subgroups stratified by age

|  | Age ≤25 | Age >25 | Total |
| --- | --- | --- | --- |
| Model A^a^ |  |  | *P*-interaction: 0.045 |
| One line slope, β (95%CI) *P*-value | -0.044 (-0.067, -0.021) 0.0002 | -0.038 (-0.050, -0.026) <0.0001 | -0.039 (-0.050, -0.028) <0.0001 |
| Model B^b^ |  |  | *P*-interaction: 0.04 |
| Turning point (K), μg/m^3^ | 38.1 | 38.3 | 38.28 |
| < K, β (95%CI) *P*-value | -0.55 (-0.84, -0.26) 0.0002 | -0.76 (-0.90, -0.61) <0.0001 | -0.71 (-0.84, -0.58) <0.0001 |
| > K, β (95%CI) *P*-value | -0.043 (-0.066, -0.021) 0.0002 | -0.037 (-0.050, -0.025) <0.0001 | -0.038 (-0.049, -0.027) <0.0001 |
| Slope 2 – Slope 1, β (95%CI) *P*-value | 0.51 (0.21, 0.80) 0.0007 | 0.72 (0.57, 0.87) <0.0001 | 0.67 (0.54, 0.80) <0.0001 |
| Predicted 25OHD levels at K (95% CI), ng/mL | 20.35 (20.01, 20.69) | 21.11 (20.93, 21.29) | 20.95 (20.79, 21.11) |
| LRT^c^, *P*-value | <0.001 | <0.001 | <0.001 |

Adjusted for year, season, 60-day moving daily average atmospheric pressure, sunshine duration, relative humidity and wind speed.

^a^Linear analysis, *P*-value <0.05 indicates a linear relationship.

^b^Non-linear analysis.

^c^*P* <0.05 means Model B is significantly different from Model A, which indicates a non-linear relationship.

Abbreviations: PM_10_, particulate matter with an aerodynamic diameter of ≤10 μm; 25OHD, 25-hydroxy vitamin D; CI, confidence interval; LRT, logarithmic likelihood ratio test.
